# Evolution of parasitism genes in the plant parasitic nematodes

**DOI:** 10.1038/s41598-024-54330-3

**Published:** 2024-02-14

**Authors:** Mehmet Dayi

**Affiliations:** 1 Forestry Vocational School, Düzce University, Konuralp Campus, 81620 Düzce, Turkey; 2 Faculty of Medicine, University of Miyazaki, Miyazaki, Japan; 3 Department of Integrated Biosciences, Graduate School of Frontier Sciences, The University of Tokyo, Chiba, 277-8562 Japan

**Keywords:** Parasite evolution, Genomics

## Abstract

The plant-parasitic nematodes are considered as one of the most destructive pests, from which the migratory and sedentary endoparasitic plant parasitic nematodes infect more than 4000 plant species and cause over $100 billion crop losses annually worldwide. These nematodes use multiple strategies to infect their host and to establish a successful parasitism inside the host such as cell-wall degradation enzymes, inhibition of host defense proteins, and molecular mimicry. In the present study, the main parasitism-associated gene families were identified and compared between the migratory and sedentary endoparasitic nematodes. The results showed that the migratory and sedentary endoparasitic nematodes share a core conserved parasitism mechanism established throughout the evolution of parasitism. However, genes involved in pectin degradation and hydrolase activity are rapidly evolving in the migratory endoparasitic nematodes. Additionally, cell-wall degrading enzymes such as GH45 cellulases and pectate lyase and peptidase and peptidase inhibitors were expanded in the migratory endoparasitic nematodes. The molecular mimicry mechanism was another key finding that differs between the endoparasitic and sedentary parasitic nematodes. The PL22 gene family, which is believed to play a significant role in the molecular mechanisms of nematode parasitism, has been found to be present exclusively in migratory endoparasitic nematodes. Phylogenetic analysis has suggested that it was de novo born in these nematodes. This discovery sheds new light on the molecular evolution of these parasites and has significant implications for our understanding of their biology and pathogenicity. This study contributes to our understanding of core parasitism mechanisms conserved throughout the nematodes and provides unique clues on the evolution of parasitism and the direction shaped by the host.

## Introduction

Plant-parasitic nematodes are one of the most devastating plant pests by utilizing more than 4,000 higher terrestrial plants as the main nutrition source. To date, approximately 27,000 species have been described^1^. The feeding style (plant, fungi, bacteria, or other microbes) of plant-parasitic nematodes is an important determinant for the evolution of being facultative or obligate with their host or nutrition source. The evolution of nematodes is considered to have occurred independently four times within Nematoda^2–4^. A phylogenetic study on nematodes inferred from about 1200 full-length small subunit rDNA sequences placed plant-parasitic nematodes in four of twelve clades^5^. These are clade1 (Triplonchida), clade2 (Dorylaimida), clade10 (Aphelenchoididae), and clade12 (Tylenchida). Plant-parasitic nematodes use the stylet during interactions with their host for puncturing the host plant cell wall to extract nutrients and in some plant-parasitic nematodes to deliver secretory molecules into the host cells to develop a permanent feeding site^6,7^. Plant-parasitic nematodes are divided into two main categories by their feeding mechanisms: ectoparasitic (clade 1 and clade 2) and endoparasitic nematodes (clade 10 and clade 12). The endoparasitic nematodes are further divided into two sub-categories: migratory and sedentary endoparasitic nematodes.

The endoparasitic nematodes penetrate and feed within host plants. The sedentary endoparasitic nematodes such as the cyst nematodes (*Globodera* and *Heterodera* spp.) generate specialized feeding sites, which are nutritional sinks for the nematode development, by manipulating normal cell development^8^. On the other hand, the migratory endoparasitic nematodes move through they feed and disturb host cells^8^.

The migratory endoparasitic nematodes such as root lesion nematodes (*Pratylenchus* spp.), stem bulb nematode (*Ditylenchus dipsaci*), and the pine wood nematode (*Bursaphelenchus xylophilus*), and sedentary endoparasitic nematodes root-knot (*Meloidogyne* spp.) and cyst nematodes (*Globodera* and *Heterodera* spp.) are recognized the most significant and scientifically relevant plant-parasitic nematodes^8^.

The plant cell wall composition has a key role in nematode-plant interactions. The major components of plant cell walls are cellulose, hemicellulose, lignin, and pectin polymers^9^, and it is the primary barrier to any pathogen or parasite^10^. Plant-parasitic nematodes employ a wide range of mechanisms for successful infection and establishment in host plants during the interactions. The cell wall degrading enzymes such as cellulases, pectate lyase, and xylanases^11^ are segregated by both the migratory and sedentary plant-parasitic nematodes for successful entry into and survival inside the host. Another secretory protein for cell wall degradation is pectate lyases. This protein is involved in the degradation of pectin and was characterized in several sedentary and migratory endoparasitic nematodes^10,12–17^.

Peptidase and peptidase inhibitors are other proteins involved in parasitism. Peptidases are a diverse group of proteins involved in the hydrolysis of peptides into amino acids by plant parasitic nematodes^18^. These proteins have various functions in plant-parasitic nematodes, such as penetration into host tissues via digestion of host plant proteins, protection from host immune systems, molting and resorption of the cuticle, and embryonic development^19^. Peptidase inhibitors are thought to be involved in parasitism in various ways in parasitic nematodes via modulating proteolytic enzyme activity during nematode-host interactions, such as protection from the host immune system^12^. The current state of knowledge regarding the presence of peptidase inhibitors in plant-parasitic nematodes is quite limited. While there have been a few studies conducted on the topic, primarily on *B. xylophilus*^12^ and *M. incognita*^20^ there is still much to learn about the peptidase inhibitor repertoire of these nematodes. In fact, the majority of the peptidase inhibitor content of plant-parasitic nematodes remains unknown. Further research is needed to gain a comprehensive understanding of the peptidase inhibitor content and evolution within plant-parasitic nematodes. By uncovering this information, we can more effectively manage and control these parasitic nematodes, which can cause significant damage to plants and crops.

A huge amount of genomic and transcriptomic data about the plant-parasitic nematodes has accumulated thanks to recent advancements in Next Generation Sequencing (NGS) technologies. This data opened new gateways for comparative genomic studies to uncover shared and unique features in the evolution of parasitism mechanisms used by the plant-parasitic nematodes.

In the present study, the most economically important migratory and sedentary endoparasitic nematodes from clades 10 and 12 were used to characterize and compare the main gene families involved in nematode-host interactions during parasitism.

## Results

### Species tree and ortholog gene relationships

**Figure 1:**
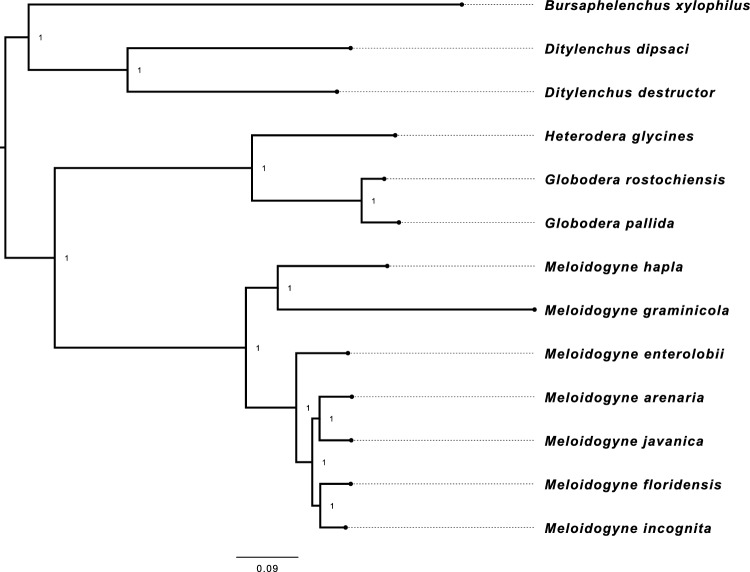
The Phylogenetic Tree of the Plant-Parasitic Nematodes. The phylogenetic tree was generated on 2084 orthogroups, each with at least 23.1% of species containing single-copy genes. The tree was rooted at the midpoint. The nematode species were divided into two distinct clusters. The migratory endoparasitic nematodes perform a separate clade from the sendentary endoparasitic nematodes.

The phylogenetic analysis of nematodes has revealed that they can be classified into two distinct clades (Fig. 1). The first clade comprises the migratory endoparasitic nematodes, while the second clade is composed of the sedentary endoparasitic nematodes. These two clades could have evolved differently, and the analysis suggests that their feeding category, or host-nematode interactions, may have played a crucial role in their genome evolution.

**Figure 2:**
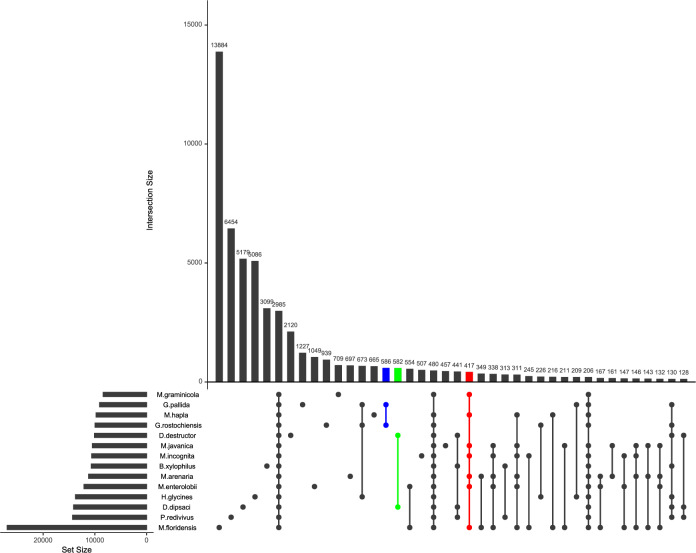
The Distribution of Genera- and Species-Specific Ortholog Families in The Plant-Parasitic Nematodes.2985 ortholog families were shared between nematode species. The highest number of species-specific ortholog families (13,864) were detected in *M. floridensis*, while the lowest number of species-specific ortholog families (457) were detected in *M. javanica*.

Ortholog gene analyses in the plant-parasitic nematodes highlighted genus- and species-specific gene families (Fig. 2). The highest number of genus-specific ortholog genes was found in the genus *Globodera* (586), followed by *Ditylenchus* (582) and *Meloidogyne* (417) (Fig. 2). On the other hand, species-specific ortholog gene numbers greatly varied among species. *M. floridensis* has the highest number of species-specific ortholog genes (13,884), while *B. xylophilis* has the lowest number of species-specific ortholog genes (3099) (Fig. 2).

Species-specific gene families were involved in various functions in species. Parasitism-associated genes, such as peptidases, were enriched in the migratory endoparasitic nematodes: *B. xylophilus* (Figure S1) and *D. dipsaci* (Figure S1). On the other hand, in the sedentary endoparasites species-specific ortholog genes were involved in various biological processes such as DNA polymerase activity and DNA biosynthetic process in *G. pallida* (Figure S1) transmembrane transport activity in *G. rostochiensis* (Figure S1), protein catabolic process in *H. glycines* (Figure S1), carboxylic acid metabolic process in *M. arenaria* (Figure S1), fibrillar center and apical protein localization in *M. enterolobii* (Figure S1), regulation of protein polyubiquitination in *M. floridensis* (Figure S1), terpenoid process in *M. graminicola* (Figure S1) and tRNA activity and histone demethylase in *M*. *hapla* (Figure S1).

### Gene family evolution

**Figure 3:**
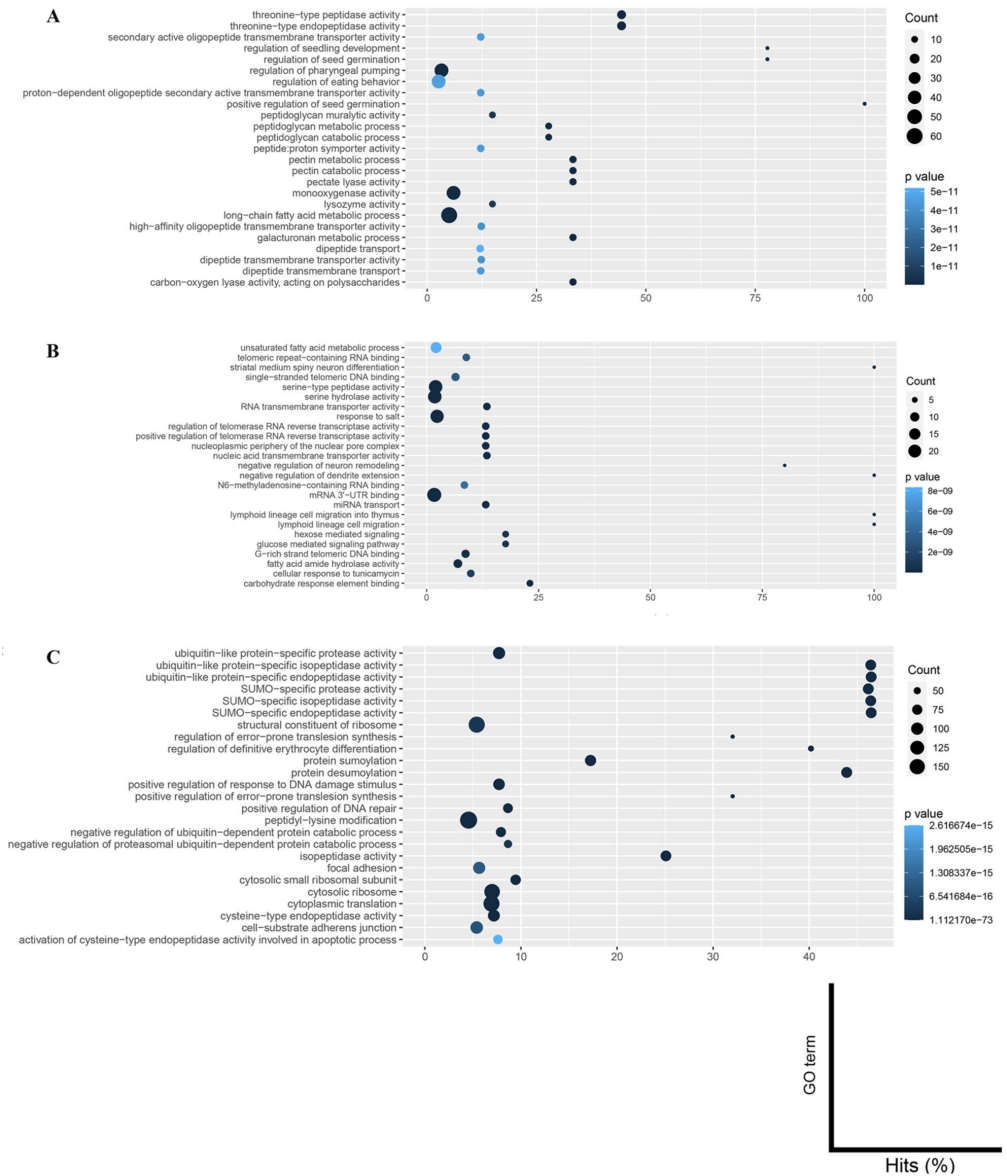
The Top 25 Annotation Results of Rapidly Evolving Gene Families Involved in Parasitism in The Migratory Endoparasitic Nematodes Compared to the Sedentary Endoparasitic Nematodes. (**A**) *Bursaphelenchus xylophilus*. (**B**) *Ditylenchus destructor*. (**C**) *Ditylenchus dipsaci*.

The study found that the gene families associated with parasitism were only detected and enriched in the migratory endoparasites' rapidly evolving gene family (Fig. 3). The CAFÉ v2.0^21^ tool calculates exact p-values for transitions between the parent and child family sizes for all phylogenetic tree branches (https://hahnlab.github.io/CAFE/src_docs/html/Report.html). A low p-value indicates a rapidly evolving branch. Rapidly evolving genes are characterised by positive selection, pseudogenization, and tandem gene arrays^22^. These genes may be the most crucial players in the host–pathogen interaction and the pathogen’s specialization on new hosts^23^. The genes involved in evading host defenses or developing novel mechanisms of infection, such as the production of novel toxins, may fall under this category^24^. The findings suggest that these genes could be involved in the evolution of parasitism.

Therefore, only these results are given in the present study (Table 1). No parasitism-associated gene families were enriched in the sedentary endoparasitic nematodes' rapidly evolving gene family category.

**Table 1:** The Distribution of Rapidly Evolving Gene Families in The Plant-Parasitic Nematodes.

The Plant-Parasitic Nematode Species	Number of Rapidly Evolving Gene Families
*Bursaphelenchus xylophilus*	181
*Ditylenchus destructor*	127
*Ditylenchus dipsaci*	960
*Globodera pallida*	983
*Globodera rostochiensis*	582
*Heterodera glycines*	1140
*Meloidogyne arenaria*	4358
*Meloidogyne enterolobii*	2256
*Meloidogyne floridensis*	4031
*Meloidogyne graminicola*	159
*Meloidogyne hapla*	348
*Meloidogyne incognita*	3694
*Meloidogyne javanica*	1923

Genes involved in peptidoglycan and catabolic processes, pectin metabolic processes, pectin catabolic processes, and pectate lyase activity were enriched in *B. xylophilus* (Fig. 3A and Table S1). On the other hand, peptidase or peptidase activity-associated gene families such as serine-type endopeptidase activity and serine hydrolase activity were enriched in *D. destructor* (Fig. 3B and Table S2), and genes involved in SUMO-specific protease activity, SUMO-specific isopeptidase activity, SUMO-specific endopeptidase activity, and cysteine-type endopeptidase activity were enriched in *D. dipsaci* (Fig. 3C and Table S3).

The study involved a comparative analysis of the rapidly evolving gene families found in migratory and sedentary endoparasitic nematodes. The analysis revealed that approximately 700 genes associated with parasitism were more prevalent in migratory endoparasitic nematodes (Table S4). These genes were largely made up of peptidases and effectors. The enriched gene families in migratory endoparasitic nematodes primarily involved pectate lyase, pectin catabolism and metabolism, and peptidoglycan catabolism and metabolism (Table S4). These processes are crucial for the nematodes to penetrate the host's tissues, evade host immune responses, and extract nutrients from the host.

### Peptidases and peptidase inhibitors

Four main peptidase families (Aspartic, Cysteine, Metallo, and Serine) were identified in the plant-parasitic nematodes. The total peptidase number was higher in the migratory endoparasites compared to sedentary endoparasitic nematodes (Table S5). Among identified peptidases pepsin, papain, and proly oligopeptidase were the most abundant peptidases in the plant-parasitic nematodes (Table S5). The highest number of peptidases were detected in *B. xylophilus* (246) and the lowest was in *M. javanica* (16) (Table S5). The Peptidase repertoire of *B. xylophilus* was dominated by pepsin, papain, proly oligopeptidase, and neprilysin (Table S5).

Additionally, some peptidase families were found to be genera-specific. For instance, the cysteine family peptidase C13, also known as legumain, was missing in *Meloidogyne* and *Globodera* species. Additionally, the metallo family peptidase, Aminopeptidase N was missing in *Meloidogyne* and *Heterodera* species (Table S5).

**Figure 4:**
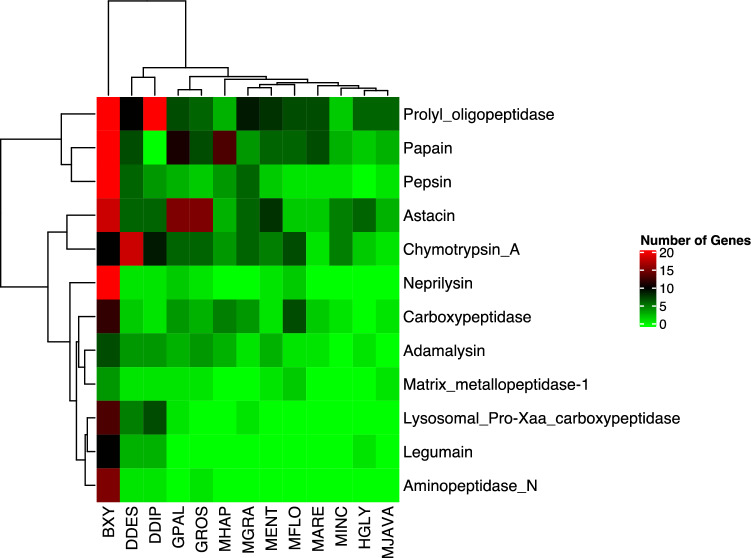
The Correlation Analysis of The Main Peptidase Families in The Plant-Parasitic Nematodes. BXY: *Bursaphelenchus xylophilus*, DDES: *Ditylenchus destructor*, DDIP: *Ditylenchus dipsaci*, GPAL: *Globodera pallida*, GROS: *Globodera rostochiensis*, HGLY: *Heterodera glycines*, MARE: *Meloidogyne arenaria*, MENT: *Meloidogyne enterolobii*, MFLO: *Meloidogyne floridensis*, MGRA: *Meloidogyne graminicola*, MHAP: *Meloidogyne hapla*, MINC: *Meloidogyne incognita*, MJAVA: *Meloidogyne javanica.* The nematodes were separated into two clusters based on peptidase numbers in genomes. The migratory endoparasitic nematode *B. xylophilus* performed a distinct clade from other species Other migratory endoparasites (*Ditylenchus* spp.) were placed as a sister clade to sedentary endoparasites.

The Pearson Correlation test was conducted on peptidases to assess the relationships amongst plant-parasitic nematodes. The resulting analysis placed the nematodes into two primary clades, as depicted in Fig. 4. The first clade consists of sedentary endoparasites, while the second clade includes migratory endoparasites. Notably, *B. xylophilus* was found to be distinct from all other species, forming its own clade (as seen in Fig. 4). Furthermore, the migratory endoparasites (Ditylenchus spp.) were placed in a sister clade to the sedentary endoparasites (as depicted in Fig. 4).

A total of nine peptidase inhibitor families were identified in the plant-parasitic nematodes. Those include I63, I2, I43 (oprin), I8, I25B, I15 (antistasin), I1, I93 and I33 (aspin-2) (Table S6). These inhibitors inhibit a wide range of peptidases released by host plants during host-parasite interactions^25^.

Plant-parasitic nematodes have a considerable amount of peptidase inhibitors (Table S8). Among them, the I63 family peptidase inhibitors are the most abundant, and they specifically target pappalysin-1 peptidase in host plants^25^. The I2 peptidase inhibitor family is the second most abundant and inhibits S1 (trypsin, chymotrypsin, elastase), and S8 (subtilisin) peptidases of host plants. These inhibitors could help the nematodes to survive and thrive by preventing the breakdown of important proteins that are necessary for their growth and development^25^.

**Figure 5:**
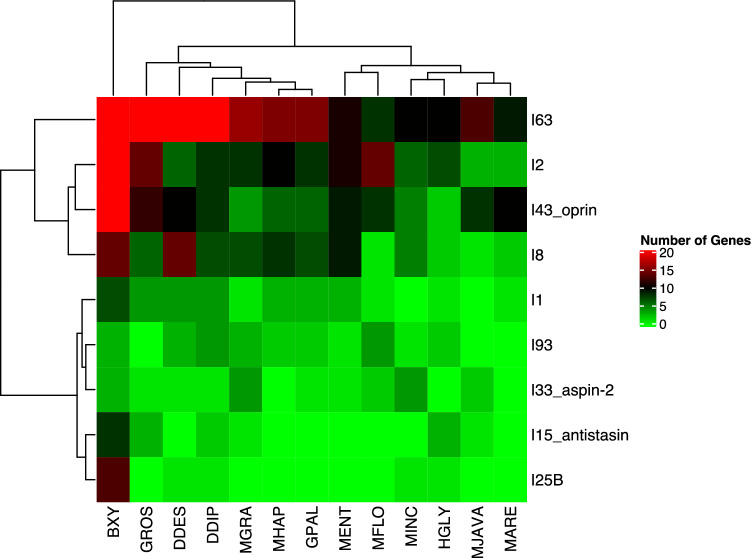
The Correlation Analysis of The Main Peptidase Inhibitor Families in The Plant-Parasitic Nematodes. BXY: *Bursaphelenchus xylophilus*, DDES: *Ditylenchus destructor*, DDIP: *Ditylenchus dipsaci*, GPAL: *Globodera pallida*, GROS: *Globodera rostochiensis*, HGLY: *Heterodera glycines*, MARE: *Meloidogyne arenaria*, MENT: *Meloidogyne enterolobii*, MFLO: *Meloidogyne floridensis*, MGRA: *Meloidogyne graminicola*, MHAP: *Meloidogyne hapla*, MINC: *Meloidogyne incognita*, MJAVA: *Meloidogyne javanica.* The Pearson Correlation test based on the number of peptidase inhibitors in the plant-parasitic nematodes separated *B. xylophilus* from other species and placed this species as a separate clade.

The total number of peptidase inhibitors in the plant parasitic nematodes showed a similar trend with peptidase numbers. These inhibitors were dominant in the migratory endoparasite species (Table S6). The highest number of peptidase inhibitors were detected in *B. xylophilus* (126), followed by *G. rostochiensis* (64), and *D. destructor* (61), while the lowest number was detected in *M. arenaria* (25) (Table S6). Additionally, species-specific peptidase inhibitor expansion was observed. For example, the I25 family peptidase inhibitors which target C1 and legumain peptidases in host plants^12^, was expanded in *B. xylophilus* (Table S6). The Pearson Correlation test based on the number of peptidase inhibitors in the plant-parasitic nematodes clearly separated *B. xylophilus* from other species and placed this species as a separate clade (Fig. 5).

### The plant cell wall degrading enzymes (PCWDE) and other effectors

The PCWDE families were classified based on enzyme–substrate into 11 gene families as ligno-cellulases (GH3, GH4, GH45, GH27, GH31, GH35, GH43, GH47, and GH99) and pectinases (PL3 and PL22).

The total number of PCWDE greatly varied among the plant-parasitic nematodes. The highest number of PCWDE was observed in *M. javanica* (114) and *D. dipsaci* (114), followed by *M. arenaria* (106), while the lowest number of PCWD was observed in *G. rostochiensis* (31) (Table S7).

The number of PCWDEs encoding ligno-cellulases ranged from 21 to 71. The highest number of ligno-cellulases was observed in *M. javanica*, while the lowest number of ligno-cellulases was observed in *D. destructor*. GH31, GH35, and GH47 were the most dominant ligno-cellulase enzymes found in all species (Table S7). On the contrary, species-specific ligno-cellulases expansions were observed. For instance, GH45 (also named Endoglucanase, endo-β-1,4-glucanase, cellulase), was dominant in *B. xylophilus* and was absent in other species except *M. floridensis*.

**Figure 6:**
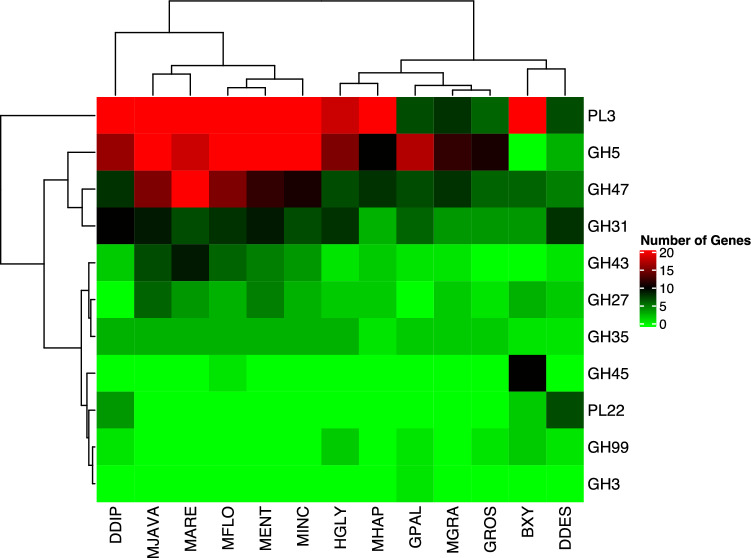
The Distribution of Plant Cell Wall Degrading Enzymes in The Plant-Parasitic Nematodes. BXY: *Bursaphelenchus xylophilus*, DDES: *Ditylenchus destructor*, DDIP: *Ditylenchus dipsaci*, GPAL: *Globodera pallida*, GROS: *Globodera rostochiensis*, HGLY: *Heterodera glycines*, MARE: *Meloidogyne arenaria*, MENT: *Meloidogyne enterolobii*, MFLO: *Meloidogyne floridensis*, MGRA: *Meloidogyne graminicola*, MHAP: *Meloidogyne hapla*, MINC: *Meloidogyne incognita*, MJAVA: *Meloidogyne javanica.* The PL3, Pectate Lyase, is one of the most significant contributors to clustering enzymes in the nematode species and is abundant in the root-knot nematodes (*Meloidogyne* species) compared to other nematode species. This family performed a separate clade from other cell-wall degrading enzymes in the nematode genomes.

In addition to ligno-celluases number of pectinases also varied among the plant-parasitic nematodes. The highest number of pectinases was observed in *D. dipsaci* (74), followed by *M. arenaria* (45) and *M. javanica* (43). Based on the results of the Pearson Correlation test, it was found that there was a significant variation in the PL3 numbers in the plant-parasitic nematodes when compared to other PCWDEs, as indicated in Fig. 6. This indicates that the PL3 family may been evolved for host specificity and host defense system, therefore could affect the pathogenicity of the nematodes. It has also been reported that *M. arenaria* and *M. javanica* have a unique genome ploidy characteristic^26^, in which they possess a triplicated genome. This presence of alleles could affect their PCWDE content, ultimately affecting their pathogenicity. Therefore, it is important to consider the impact of genome ploidy and the presence of alleles on the pathogenicity of nematodes when studying their PCWDE content.

**Figure 7:**
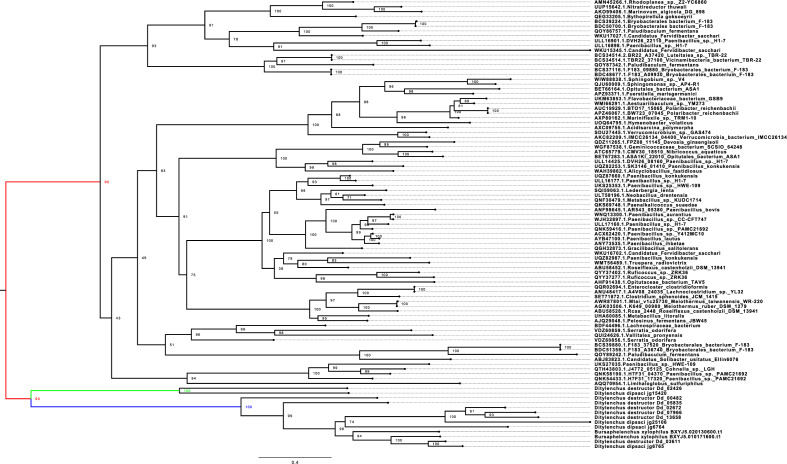
The Phylogenetic Tree of the PL22 (Oligogalacturonate lyase/oligogalacturonide lyase). The tree was generated using IQ-TREE v2.0 with -m MFP (model finder parameter) with -B 1000 (1000 bootstrap) options. The tree was separated into two clades. The black branch represent Bacteria clade, and the red branch represents nematodes. The green and blue branches show two differenct clades of the PL22 genes detected in the migratory endoparasitic nematodes (*Bursaphelenchus xylophilus*, *Ditylenchus destructor* and *Ditylenchus dipsaci*). Numbers on nodes represent bootstrap values. The nematode PL22 genes showed a clear pattern that this gene family could be a eukaryote origin.

The PL22 pectinase family was detected only in the migratory endoparasitic nematodes. This family was absent in the sedentary endoparasites (Table S7). Further research was performed to identify the possible origin of the PL22 family in the migratory endoparasitic nematodes. It was found that this family was eukaryotic origin and PL22 genes of the migratory endoparasitic nematodes were performed a distinct clade from those in Bacteria based on the phylogenetic analysis (Fig. 7). The phylogenetic tree suggested that this family resulted from a de novo gene birth, followed by gene duplication events in the migratory endoparasitic nematodes.

Plant-parasitic nematodes have been identified to contain several effectors to date. These effectors have been characterized and identified as HYP^27^, SPRYSEC^28,29^, SPRY^30,31^, expansin^32^, Cathepsin_L^33,34^, CLAVATA3/ESR_(CLE)^35^ and annexin^36,37^. A blastp^38^ search was conducted with a cutoff of 1e-10 on the protein sequences of these effectors downloaded from from UniProt^39^ to find orthologs of these effectors in other plant-parasitic nematodes. Cathepsin_L, SPRY and annexin were detected in all nematode species with different numbers, as shown in Table S8. Interestingly, the SPRYSEC effector was only detected in the cyst nematodes (Globodera and Heterodera species) (Table S8), as mentioned in a review by Diaz-Granados et al.^40^, this point was also highlighted^40^. These findings suggest that different nematode species may have their unique set of effectors. The presence of SPRYSEC effectors exclusively in cyst nematodes points towards their distinctiveness. These effectors may have a significant impact on the pathogenic traits of these nematodes, which further highlights their unique genetic makeup and characteristics.

### Putative proteins involved in molecular mimicry for evasion from the host defense

In the plant-parasitic nematodes several parasitism-associated gene families have the best significant hit with orthologs of plant genes and no significant best hit to any nematode species were identified (Table S9). These are peptidases and peptidase inhibitors belonging to cysteine (C1, C19, and cystatin), aspartic (A1, pepsin), serine peptidases (Carboxypeptidase and proly oligopeptidase), and peptidase inhibitors (I29 and serpin) (Table S9). In *B. xylophilus* cystain family inhibitors were detected. In *Ditylenchus* species peptidases belonging to A1 and C1 families and prolyl endopeptidases and serpin peptidases were detected (Table S9). In *Meloidogyne* species peptidases belonging to the cysteine family (C1 and C10) and serine family (S10) were detected. Other protein families involved in parasitism in the plant-parasitic nematodes were also detected as best hit with plant orthologs. These included SPRY and Thaumatin domain-containing proteins and were seen only in *Globodera* and *Heterodera* species (Table S9).

## Discussion

In the present study, comparative genomic analyses on the main parasitism mechanisms of the most economically and scientifically critical plant-parasitic nematodes belonging to clade 10 and clade 12 were performed. To the best of my knowledge, this study provided a broader perspective on the main gene families and their evolution which are involved in host-parasite interactions in thirteen plant-parasitic nematodes from five genera and two clades. The host range and feeding strategy of the plant-parasitic nematodes used in the present study are largely diverse. For example, the migratory endoparasitic nematodes *B. xylophilus,* which belongs to clade 12, feeds on pine species, while other plant-parasitic nematodes prefer agricultural plants^8^.

The comparative analyses of main parasitism genes have provided insights into common versus divergent features and numbers among the plant-parasitic nematodes. Even though species share common genes such as peptidases and peptidase inhibitors, cellulases, pectate lyases involved in parasitism, which explains that the plant-parasitic nematodes share a conserved parasitism system throughout evolution, that encompasses the requirements for successful infection and overcome host defense, the global distribution of genes encoding parasitism-associated genes varied. This variation could be a result of the feeding strategy and host species. For example, the correlation analysis of peptidase and peptidase inhibitors highlighted the pine wood nematode as a separate clade from other parasitic nematodes. The A1 (Pepsin) and the C1 (papain family) peptidases are one of the determinants of this separation. These peptidases were also found to be expanded via intensive gene duplication in a previous study^12^.

The I25B peptidase inhibitor was the primary determinant for the correlation of the peptidase inhibitors in the plant-parasitic nematodes. The I25B family contains proteins inhibiting C1 family peptidases and legumain, which play various physiological roles in plant defense. The pine wood nematode prefers higher plants (pines) as hosts distinct from host plants of other nematodes investigated in the present study.

The pine wood nematode is an exceptional species because of expansions in nearly all peptidase and peptidase inhibitors among the species studied, which supports the previous study^12^. Once the pine wood nematode enters a pine tree, it migrates through the resin canals of the tree by destroying and feeding on the parenchymal cells^12^. The resin canals are absent in host species of other plant-parasitic nematodes. Therefore, host evolution also could be a key factor for the evolution of parasitism in plant parasitic nematodes. We, therefore, could speculate that the global distribution of peptidase families is somehow associated with plant cell wall characteristics.

Genes encoding peptidases and peptidase inhibitors were found to be expanded in the migratory endoparasitic nematodes compared to those in the sedentary endoparasitic nematodes. This reflects peptidases and peptidase inhibitors are key proteins for successful establishment inside the host and move throughout host and overcoming the host defense. Another key funding suggests that peptidases of the migratory endoparasitic nematodes are greatly divergent from those in the sedentary parasitic nematodes.

Plant-parasitic nematodes secretes proteins that are able to alter the plant's defense mechanisms in a way that protects nematodes from molecules such as the reactive oxygen species produced by the plant in response to the nematode's parasitic attack^41^. Migratory endoparasitic nematodes move inside their host as they feed. Due to their active movement, they could be differently exposed to host defense compared to sedentary endoparasitic nematodes. However, these nematodes can overcome the host's defense system by mimicking the host's defense peptidases^7^. Various peptidase families are involved in parasite-host interactions, and there is a sequence similarity between these peptidases in migratory endoparasitic nematodes and those found in plants. In particular, the C1 peptidase (papain family) of migratory endoparasitic nematodes bears the highest level of similarity to those found in plants.

Interestingly, C1 peptidases play various roles in plants, such as defense against pathogenic microbes and herbivorous arthropods^42^. This suggests that the C1 peptidases in migratory endoparasitic nematodes may serve a similar function in overcoming the host's defense system. The C1 peptidases family could regulate immunomodulation and inhibit the host's immune system, thus protecting the nematode from the potent effects of host immune system. The results of this study offer a comprehensive understanding of how migratory endoparasitic nematodes are able to outsmart the host's immune system and successfully infect their host. The study highlights the complex mechanisms employed by these parasites to evade detection, including the use of molecular mimicry to disguise themselves as host cells, the secretion of immunomodulatory molecules to suppress the host's immune response, and the ability to rapidly adapt to different host environments. These insights not only deepen our knowledge of host-parasite interactions but also have important implications for the development of new strategies to control nematode infections in plants.

The tight association between the plant-parasitic nematodes and the host is a driving force in the evolution of parasitism and accelerates the evolution of parasitism-associated gene families in the plant-parasitic nematodes. It was found that the pine wood nematode genes encoding pectin degradation, peptidoglycan metabolic, and catabolic processes are rapidly evolving compared to other genes. The secretory protein, pectate lyase (PL3), is involved in the breakdown of pectin that supports the cellulose and hemicellulose fiber molecules within and between plant cell-walls^43^, and allows nematodes migratory through the intercellular spaces^43^. This protein was found to be rapidly evolving in the pine wood nematode. This suggests that the pine wood nematode pectate lyase genes are under strong evolutionary pressure during the nematode-host interactions, and the PL3 could be an insight into the direction of evolution in parasitism in the plant-parasitic nematodes.

The present study has produced an interesting finding that sheds light on the PL22 family of plant-parasitic nematodes. It has been discovered that this particular family is exclusively found in the migratory endoparasitic nematodes, and is absent in sedentary parasites. This information provides valuable insights into the evolutionary history of parasitic nematodes. Further research could help us understand the mechanisms behind this family's ability to thrive in migratory endoparasitic nematodes and its absence in sedentary ones. The phylogenetic analysis conducted on migratory endoparasitic nematodes has revealed an interesting finding. The analysis suggests that these nematodes might have undergone a de novo gene birth event, which led to the creation of a gene that encodes the PL22 family. This gene family might play a crucial role in the development and survival of these nematodes inside the host. The de novo gene birth event is a fascinating process that occurs when new genes evolve from DNA sequences that were not originally genic. These genes can either code for proteins or act as RNA genes. It is considered a crucial mechanism in the evolution of new genes^44^. The discovery of this event in migratory endoparasitic nematodes is significant because it provides new insights into the evolution of nematodes and the mechanisms involved in creating novel genes. In particular, it helps us understand how nematodes have evolved over time and how they have adapted to their environment.

The study of this event in migratory endoparasitic nematodes has revealed that the process is more complex than previously thought. It involves a combination of mechanisms, including de novo gene birth and gene duplication. Although this is a hypothetical assumption with the support of the phylogenetic analysis and requires a deep and comparative evolutionary study, we could speculate that the de novo gene birth could be another mechanism effecting evolution of parasitism in the plant-parasitic nematodes. Overall, the discovery of de novo gene birth events in migratory endoparasitic nematodes is a significant breakthrough in our understanding of the evolution of new genes. It provides new insights into the mechanisms involved in creating novel genes and could have implications for the evolution of other organisms as well. The analysis also revealed multiple duplications in this family after the de novo gene birth in the migratory endoparasitic nematodes. These duplications led to the expansion of the PL22 family and the emergence of new proteins that might have unique or species-specific functions. The study of these proteins could provide insights into the adaptation of migratory endoparasitic nematodes to their hosts and the evolution of parasitism in nematodes.

Glycoside hydrolases and polysaccharide lyases (PL) are enzymes involved in the carbohydrate metabolic process; some are known as cell-wall degrading enzymes. The GH45 family (cellulases) was found intensively in the pine wood nematode, which is agreed in a previous study^10,12,45^, and is well documented that the pine wood nematode has acquired cellulases via horizontal gene transfer from fungi during the evolution of parasitism by nematodes^46^. This suggest that the pine wood nematode has a unique mechanism for cell wall degradation. We could see similar unique feature of evolution of cell wall degrading enzymes because the pectate lyase was found to be under rapidly evolving in *B. xylophilus* as mentioned above.

To survive and thrive, plant-parasitic nematodes employ a strategy called molecular mimicry. This strategy involves the secretion of proteins similar in structure to those found in the host plant, which helps the nematodes avoid being detected by the host’s immune system. One example of molecular mimicry in nematodes is seen in cyst nematodes^47^. These nematodes secrete proteins that contain a conserved 12-amino acid C-terminal motif, which is highly similar to plant CLAVATA3/ESR (CLE) ligand peptides^48^. Similarly, migratory endoparasitic nematodes like *B. xylophilus* have been found to produce thaumatin-like proteins and a cystain-like peptidase inhibitor^12,49^, which agreed with the present study. Recent studies have identified several proteins in plant-parasitic nematodes that could mimic host plant peptidase and peptidase inhibitors. These include peptidase^50^ and peptidase inhibitors^51,52^, thaumatin-like proteins^12^, and SPRY domain-containing proteins^30,53^. The long-term co-evolution between plant-parasitic nematodes and their hosts has led to the development of various mechanisms that use different proteins to overcome and escape the host defense system^7^. It is worth delving into the details of the fact that all plant-parasitic nematodes use molecular mimicry to evade the host defense pathogen detection system. However, the specific mechanisms employed by each species differ. This could be due to a long-term co-evolution between the plant-parasitic nematodes and their hosts, which has influenced the development of various strategies employed by these nematodes to overcome and evade the host defense system.

It is crucial to comprehend the particular strategies employed by different nematodes, as this knowledge can help in the development of more effective methods of controlling plant parasitic nematodes. Understanding how these nematodes manipulate their host plants can also help in the development of more resilient crops that can resist nematode infection.

## Materials and methods

### Data set

Protein files of each plant-parasitic nematodes species were downloaded from wormbase^54^ (Table 2). Only the plant-parasitic nematode species that have proteome files derived from whole genome sequencing were considered in the present study (Table 2).

**Table 2:** The Plant-Parasitic Nematodes and their genomic features used in the present study.

The Plant-Parasitic Species	Common Name	Feeding Type	Assembly Accession	Genome Size (Mbp)	Number of Protein Coding Gene (n)
*Bursaphelenchus xylophilus*	The pinewood nematode	Migratory endoparasite	GCA_904067135.1	78,2	15,884
*Ditylenchus destructor*	Potato rot nematode	Migratory endoparasite	GCA_001579705.1	111,1	13,931
*Ditylenchus dipsaci*	Stem nematode	Migratory endoparasite	GCA_004194705.1	227,2	26,428
*Globodera pallida*	Potato cyst nematode	Sedentary endoparasite	GCA_000724045.1	123,6	16,403
*Globodera rostochiensis*	Golden nematode	Sedentary endoparasite	GCA_900079975.1	95,8	14,308
*Heterodera glycines*	Soybean cyst nematode	Sedentary endoparasite	GCA_004148225.1	123,8	29,679
*Meloidogyne arenaria*	Peanut rot nematode	Sedentary endoparasite	GCA_003693565.1	163,7	27,548
*Meloidogyne enterolobii*	Guava root-knot nematode	Sedentary endoparasite	GCA_003693675.1	162,9	29,473
*Meloidogyne floridensis*	Peach root-knot nematode	Sedentary endoparasite	GCA_000751915.1	96,6	49,938
*Meloidogyne graminicola*	Rice root-knot nematode	Sedentary endoparasite	GCA_002778205.1	38,1	10,895
*Meloidogyne hapla*	Northern root-knot nematode	Sedentary endoparasite	GCA_000172435.1	53,0	14,419
*Meloidogyne incognita*	Southern root-knot nematode	Sedentary endoparasite	GCA_900182535.1	183,5	43,718
*Meloidogyne javanica*	Sugarcane eelworm	Sedentary endoparasite	GCA_003693625.1	150,3	24,679

### Ortholog gene relationships

OrthoFinder v2.2.6^55^ was used to identify ortholog relationships (shared and species-specific) among the plant-parasitic nematodes with -M msa. The species tree was generated using STAG^56^ implemented in OrthoFinder v2.2.6^55^. Species-specific ortholog families were further extracted from OrthoFinder v2.2.6^55^ results and were visualized using upsetR R package^57^. Functional annotation of these ortholog families was performed using eggNOG v5.0^58^ database. Annotation results were further analyzed using GOseq v.144.0 R package^59^ to identify enriched GO terms.

### Gene family evolution

Proteins of the plant parasitic nematodes were clustered into ortholog families using OrthoFinder v2.2.6^55^. The phylogenetic tree (Fig. 1) generated during ortholog analysis was converted to an ultrametric tree using the r8s program v1.81^60^. CAFE v2.0^21^ was then run to estimate the birth–death parameter λ (the rate of change of evolution in a tree) (https://hahnlab.github.io/CAFE/src_docs/html/Lambda.html)of gene family clusters with the ultrametric tree with 0.01 p-value threshold, and the estimated λ value (0.00353786) was used at the same run to detect expanded, contracted, and rapidly evolving gene families among species. Functional annotation of gene family analysis results was performed using eggNOG v5.0^58^ database. Annotation results were further analysed using GOseq v.144.0 R package^59^ to identify enriched GO terms.

### Peptidase and peptidase inhibitors

Proteins of each species were searched against peptidase and peptidase protein HMM profile files downloaded from MEROPS^25^ database using hmmsearch v3.1b1^61^ with 1e-5 cuttoff. Results were further subject to SignalP v5.0^62^ to detect signal peptides. Proteins that have signal peptides were kept and used for peptidase and peptidase inhibitor comparisons.

### Plant cell wall degrading enzymes

The CAZy HMM profiles were downloaded from the CAZy database^63^, and each species’ proteins were searched against the CAZy HMM profiles using hmmsearch v3.1b1^61^ with 1e-5 e-value cutoff.

### Identification of putative proteins involved in molecular mimicry

eggNOG-mapper v5.0^58^ that uses orthology assignment against the eggNOG database composed of precomputed clusters and phylogenies and assigs genes for each gene ontology was used to identify proteins of each species that could mimic host plants during host-parasite interactions. eggNOG v5.0^58^ functional annotation results were manually checked and the proteins that had a significant top hit to plant genes and no significant hits to nematodes were considered as potential proteins involved in mimicry against the host plants.

### Supplementary Information

Supplementary Information 1.

Supplementary Information 2.

Supplementary Information 3.

Supplementary Information 4.

Supplementary Information 5.

Supplementary Information 6.

Supplementary Information 7.

Supplementary Information 8.

Supplementary Information 9.

Supplementary Information 10.

Supplementary Information 11.

## Data Availability

The datasets generated during and/or analysed during the current study are available from the corresponding author upon reasonable request.
