# Supplementary figures and images for: Evolution of parasitism genes in the plant parasitic nematodes
Source: Sci Rep. 2024 Feb 14;14:3733. doi: 10.1038/s41598-024-54330-3 (PMC10866927; doi:10.1038/s41598-024-54330-3)

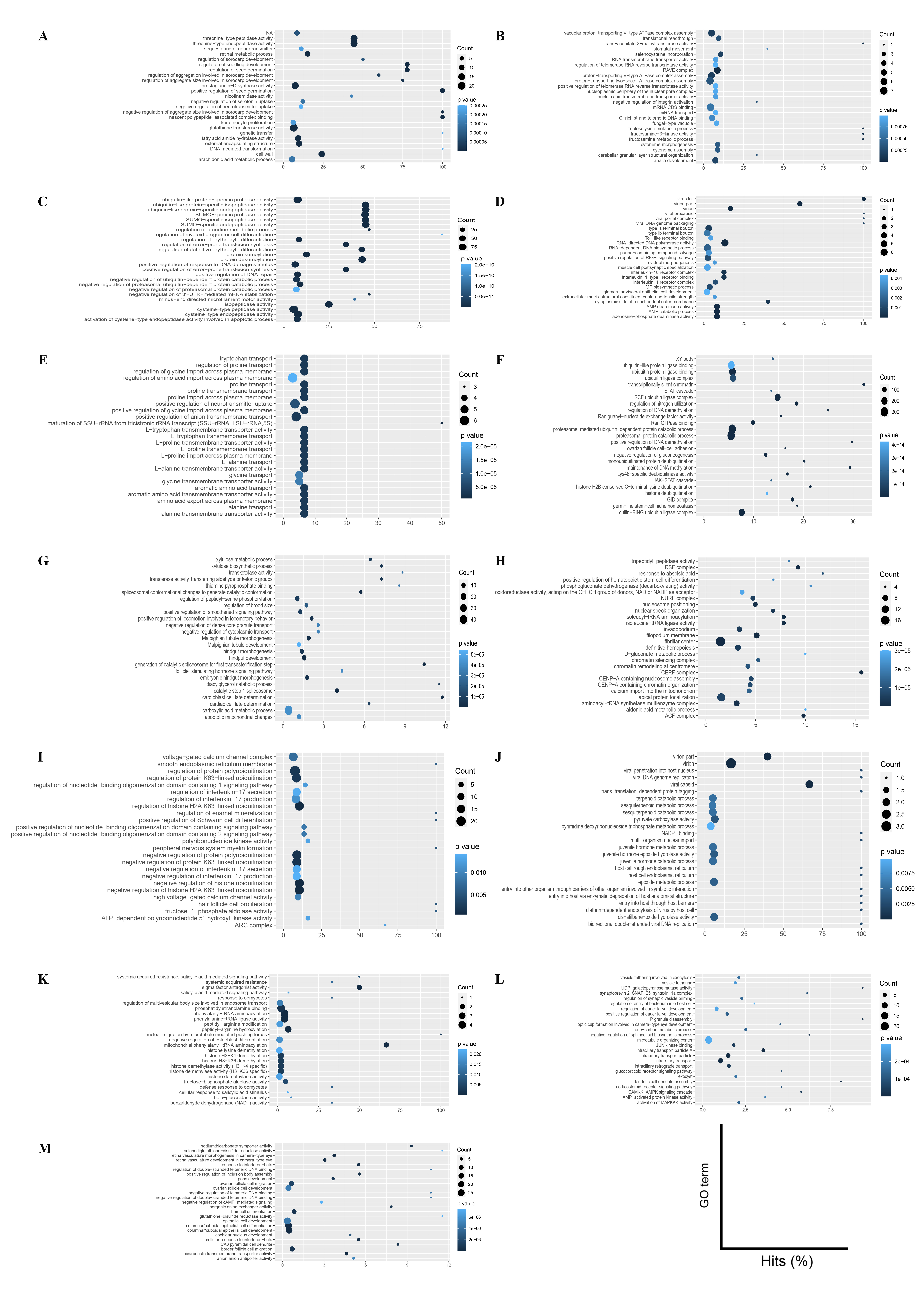

Supplement: Supplementary file 1 — Supplementary Information 1. [file 41598_2024_54330_MOESM1_ESM.jpg]
