# Supplementary material for: Evolution of parasitism genes in the plant parasitic nematodes
Source: Sci Rep. 2024 Feb 14;14:3733. doi: 10.1038/s41598-024-54330-3 (PMC10866927; doi:10.1038/s41598-024-54330-3)
Supplement: Supplementary file 6 — Supplementary Information 6. [file 41598_2024_54330_MOESM6_ESM.docx]

| **Peptidase** | | | | **Nematode Species** | | | | | | | | | | | | |
| --- | --- | --- | --- | --- | --- | --- | --- | --- | --- | --- | --- | --- | --- | --- | --- | --- |
| **Family** | | **Subfamily** | **Type enzyme** | **BXY** | **DDES** | **DDIP** | **GPAL** | **GROS** | **HGLY** | **MARE** | **MENT** | **MFLO** | **MGRA** | **MHAP** | **MINC** | **MJAVA** |
| Aspartic | A1 | A1A | Pepsin | 47 | 6 | 4 | 3 | 2 | 0 | 1 | 2 | 1 | 6 | 4 | 1 | 1 |
| Cysteine | C1 | C1A | Papain | 42 | 7 | 0 | 11 | 7 | 2 | 7 | 6 | 6 | 4 | 13 | 3 | 3 |
|  | C13 | - | Legumain | 10 | 3 | 3 | 0 | 0 | 1 | 0 | 0 | 0 | 0 | 0 | 0 | 0 |
| Metallo | M13 | - | Neprilysin | 24 | 1 | 1 | 2 | 1 | 0 | 0 | 1 | 2 | 0 | 0 | 0 | 0 |
|  | M12 | M12A | Astacin | 18 | 6 | 6 | 15 | 15 | 6 | 2 | 8 | 2 | 6 | 3 | 5 | 3 |
|  | M1 | - | Aminopeptidase N | 15 | 1 | 1 | 0 | 1 | 0 | 0 | 0 | 0 | 0 | 0 | 0 | 0 |
|  | M12 | M12B | Adamalysin | 7 | 4 | 4 | 3 | 4 | 1 | 1 | 3 | 1 | 1 | 3 | 0 | 0 |
|  | M10 | M10A | Matrix metallopeptidase-1 | 4 | 1 | 1 | 1 | 1 | 0 | 0 | 1 | 2 | 0 | 0 | 0 | 1 |
| Serine | S9 | - | Prolyl oligopeptidase | 28 | 8 | 17 | 7 | 5 | 5 | 5 | 6 | 4 | 8 | 2 | 2 | 4 |
|  | S33 | - | Prolyl oligopeptidase | 14 | 2 | 3 | 0 | 1 | 1 | 2 | 2 | 3 | 1 | 1 | 0 | 2 |
|  | S28 | - | Lysosomal Pro-Xaa carboxypeptidase | 13 | 5 | 7 | 1 | 0 | 0 | 0 | 0 | 0 | 1 | 0 | 0 | 0 |
|  | S10 | - | Carboxypeptidase | 12 | 2 | 1 | 4 | 3 | 0 | 2 | 1 | 7 | 4 | 5 | 1 | 1 |
|  | S1 | S1A | Chymotrypsin A | 10 | 18 | 9 | 6 | 6 | 2 | 1 | 5 | 7 | 6 | 4 | 5 | 1 |
|  |  |  | **Total** | **244** | **64** | **57** | **53** | **46** | **18** | **21** | **35** | **35** | **37** | **35** | **17** | **16** |

**Table S5. The Distribution of Secreted Peptidase Families in The Plant-Parasitic Nematodes^*^.** BXY: *Bursaphelenchus xylophilus*, DDES: *Ditylenchus* *destructor*, DDIP: *Ditylenchus* *dipsaci*, GPAL: *Globodera* *pallida*, GROS: *Globodera* *rostochiensis*, HGLY: *Heterodera* *glycines*, MARE: *Meloidogyne* *arenaria*, MENT: *Meloidogyne* *enterolobii*, MFLO: *Meloidogyne* *floridensis*, MGRA: *Meloidogyne* *graminicola*, MHAP: *Meloidogyne* *hapla*, MINC: *Meloidogyne* *incognita*, MJAVA: *Meloidogyne* *javanica*

*Non-peptidase homologous were not considered to count peptidases in the plant-parasitic nematodes.
