# Supplementary material for: Evolution of parasitism genes in the plant parasitic nematodes
Source: Sci Rep. 2024 Feb 14;14:3733. doi: 10.1038/s41598-024-54330-3 (PMC10866927; doi:10.1038/s41598-024-54330-3)
Supplement: Supplementary file 7 — Supplementary Information 7. [file 41598_2024_54330_MOESM7_ESM.docx]

|  | | | | | | | | | | | | | | |  |
| --- | --- | --- | --- | --- | --- | --- | --- | --- | --- | --- | --- | --- | --- | --- | --- |
|  | **Inhibitor** | **Nematode Species** | | | | | | | | | | | | | |
| **Identifier** | **Peptidases Inhibited in the Host** | **BXY** | **DDES** | **DDIP** | **GPAL** | **GROS** | **HGLY** | **MARE** | **MENT** | **MFLO** | **MGRA** | **MHAP** | **MINC** | **MJAVA** | |
| I63 | Pappalysin-1 | 38 | 22 | 20 | 15 | 24 | 10 | 9 | 11 | 8 | 16 | 15 | 10 | 13 | |
| I2 | S1 (trypsin, chymotrypsin, elastase), S8 (subtilisin) | 20 | 6 | 8 | 8 | 14 | 7 | 3 | 11 | 14 | 8 | 10 | 6 | 3 | |
| I43 (oprin) | M12 (astacin) | 20 | 10 | 8 | 6 | 12 | 2 | 10 | 9 | 8 | 4 | 6 | 5 | 8 | |
| I8 | S1 (trypsin, chymotrypsin, elastase), M4 (thermolysin) | 14 | 14 | 7 | 7 | 6 | 2 | 2 | 9 | 1 | 7 | 8 | 5 | 1 | |
| I25B | C1 and Legumain | 13 | 1 | 1 | 0 | 0 | 1 | 0 | 0 | 0 | 0 | 0 | 1 | 0 | |
| I15 (antistasin) | S1 | 8 | 0 | 2 | 0 | 3 | 3 | 0 | 0 | 0 | 1 | 0 | 0 | 1 | |
| I1 | Adamalysin | 7 | 4 | 4 | 3 | 4 | 1 | 1 | 3 | 1 | 1 | 3 | 0 | 0 | |
| I93 | M12 (astacin) | 3 | 3 | 4 | 2 | 0 | 2 | 0 | 1 | 4 | 3 | 2 | 1 | 0 | |
| I33 (aspin-2) | A1 (pepsin A, gastricsin, cathepsin E) | 3 | 1 | 1 | 1 | 1 | 0 | 0 | 1 | 2 | 4 | 0 | 4 | 2 | |
|  | **Total** | **126** | **61** | **55** | **42** | **64** | **28** | **25** | **45** | **38** | **44** | **44** | **32** | **28** | |

**Table S6. The Distribution of Secreted Peptidase Inhibitors in the Plant-Parasitic Nematodes and Their Target Peptidases Inhibited in Plants.** BXY: *Bursaphelenchus xylophilus*, DDES: *Ditylenchus* *destructor*, DDIP: *Ditylenchus* *dipsaci*, GPAL: *Globodera* *pallida*, GROS: *Globodera* *rostochiensis*, HGLY: *Heterodera* *glycines*, MARE: *Meloidogyne* *arenaria*, MENT: *Meloidogyne* *enterolobii*, MFLO: *Meloidogyne* *floridensis*, MGRA: *Meloidogyne* *graminicola*, MHAP: *Meloidogyne* *hapla*, MINC: *Meloidogyne* *incognita*, MJAVA: *Meloidogyne* *javanica*
