# Supplementary material for: Evolution of parasitism genes in the plant parasitic nematodes
Source: Sci Rep. 2024 Feb 14;14:3733. doi: 10.1038/s41598-024-54330-3 (PMC10866927; doi:10.1038/s41598-024-54330-3)
Supplement: Supplementary file 8 — Supplementary Information 8. [file 41598_2024_54330_MOESM8_ESM.docx]

| **Enzyme** | | | **Nematode Species** | | | | | | | | | | | | |
| --- | --- | --- | --- | --- | --- | --- | --- | --- | --- | --- | --- | --- | --- | --- | --- |
| Substrate | Family | Activity | BXY | DDES | DDIP | GPAL | GROS | HGLY | MARE | MENT | MFLO | MGRA | MHAP | MINC | MJAVA |
| Ligno-cellulose | GH3 | β-Glucosidases | 0 | 0 | 0 | 1 | 0 | 0 | 0 | 0 | 0 | 0 | 0 | 0 | 0 |
|  | GH5 | Endo-β-1,4-glucanase/ cellulase | 0 | 3 | 16 | 17 | 11 | 15 | 18 | 22 | 23 | 12 | 10 | 22 | 31 |
|  | GH45 | Endoglucanase, endo-β-1,4-glucanase, cellulase | 10 | 0 | 0 | 0 | 0 | 0 | 0 | 0 | 1 | 0 | 0 | 0 | 0 |
|  | GH27 | α-Galactosidases | 3 | 2 | 0 | 0 | 1 | 2 | 4 | 5 | 3 | 2 | 2 | 3 | 6 |
|  | GH31 | α-Glucosidase | 4 | 8 | 10 | 6 | 4 | 8 | 7 | 9 | 8 | 4 | 3 | 7 | 9 |
|  | GH35 | β-Galactosidases | 1 | 1 | 3 | 2 | 2 | 3 | 3 | 3 | 3 | 2 | 1 | 3 | 3 |
|  | GH43 | α-Arabinosidases | 0 | 1 | 2 | 1 | 0 | 1 | 9 | 5 | 6 | 1 | 2 | 4 | 7 |
|  | GH47 | Exo-acting α-1,2-mannosidases | 6 | 5 | 8 | 7 | 6 | 7 | 20 | 12 | 15 | 8 | 8 | 11 | 15 |
|  | GH99 | Endo-α-1,2-mannosidase | 2 | 1 | 1 | 1 | 1 | 2 | 0 | 0 | 0 | 0 | 0 | 0 | 0 |
| Pectin | PL3 | Pectate lyase | 20 | 7 | 70 | 7 | 6 | 18 | 45 | 35 | 38 | 8 | 20 | 28 | 43 |
|  | PL22 |  | 2 | 7 | 4 | 0 | 0 | 0 | 0 | 0 | 0 | 0 | 0 | 0 | 0 |
| **Total** | | | **48** | **35** | **114** | **42** | **31** | **56** | **106** | **91** | **97** | **37** | **46** | **78** | **114** |

**Table S7. The Distribution of Plant Cell Wall Degrading Enzymes in the Plant-Parasitic Nematodes.** BXY: *Bursaphelenchus xylophilus*, DDES: *Ditylenchus* *destructor*, DDIP: *Ditylenchus* *dipsaci*, GPAL: *Globodera* *pallida*, GROS: *Globodera* *rostochiensis*, HGLY: *Heterodera* *glycines*, MARE: *Meloidogyne* *arenaria*, MENT: *Meloidogyne* *enterolobii*, MFLO: *Meloidogyne* *floridensis*, MGRA: *Meloidogyne* *graminicola*, MHAP: *Meloidogyne* *hapla*, MINC: *Meloidogyne* *incognita*, MJAVA: *Meloidogyne* *javanica*
