# Supplementary material for: Evolution of parasitism genes in the plant parasitic nematodes
Source: Sci Rep. 2024 Feb 14;14:3733. doi: 10.1038/s41598-024-54330-3 (PMC10866927; doi:10.1038/s41598-024-54330-3)
Supplement: Supplementary file 11 — Supplementary Information 11. [file 41598_2024_54330_MOESM11_ESM.docx]

**Supplementary Tables**

**Table S1. The Annotation Results of Enriched Rapidly Evolving Genes in the *Bursaphelenchus xylophilus* genome.**

**Table S2. The Annotation Results of Enriched Rapidly Evolving Genes in the *Ditylenchus destructor* genome.**

**Table S3. The Annotation Results of Enriched Rapidly Evolving Genes in the *Ditylenchus dipsaci* genome.**

**Table S4. The Annotation Results of Gene Ontology Enrichment of Rapidly Evolving Genes Involved in Parasitism in the Migratory Endoparasitic Nematodes.**

**Table S5.** **The Distribution of Secreted Peptidase Families in The Plant-Parasitic Nematodes**^*^. BXY: *Bursaphelenchus xylophilus*, DDES: *Ditylenchus* *destructor*, DDIP: *Ditylenchus* *dipsaci*, GPAL: *Globodera* *pallida*, GROS: *Globodera* *rostochiensis*, HGLY: *Heterodera* *glycines*, MARE: *Meloidogyne* *arenaria*, MENT: *Meloidogyne* *enterolobii*, MFLO: *Meloidogyne* *floridensis*, MGRA: *Meloidogyne* *graminicola*, MHAP: *Meloidogyne* *hapla*, MINC: *Meloidogyne* *incognita*, MJAVA: *Meloidogyne* *javanica*

**Table S6.** **The Distribution of Secreted Peptidase Inhibitors in the Plant-Parasitic Nematodes and Their Target Peptidases Inhibited in Plants.** BXY: *Bursaphelenchus xylophilus*, DDES: *Ditylenchus* *destructor*, DDIP: *Ditylenchus* *dipsaci*, GPAL: *Globodera* *pallida*, GROS: *Globodera* *rostochiensis*, HGLY: *Heterodera* *glycines*, MARE: *Meloidogyne* *arenaria*, MENT: *Meloidogyne* *enterolobii*, MFLO: *Meloidogyne* *floridensis*, MGRA: *Meloidogyne* *graminicola*, MHAP: *Meloidogyne* *hapla*, MINC: *Meloidogyne* *incognita*, MJAVA: *Meloidogyne* *javanica*

**Table S7.** **The Distribution of Plant Cell Wall Degrading Enzymes in the Plant-Parasitic Nematodes.** BXY: *Bursaphelenchus xylophilus*, DDES: *Ditylenchus* *destructor*, DDIP: *Ditylenchus* *dipsaci*, GPAL: *Globodera* *pallida*, GROS: *Globodera* *rostochiensis*, HGLY: *Heterodera* *glycines*, MARE: *Meloidogyne* *arenaria*, MENT: *Meloidogyne* *enterolobii*, MFLO: *Meloidogyne* *floridensis*, MGRA: *Meloidogyne* *graminicola*, MHAP: *Meloidogyne* *hapla*, MINC: *Meloidogyne* *incognita*, MJAVA: *Meloidogyne* *javanica*

**Table S9.** The Putative Proteins Detected in the Plant Parasitic Nematodes that Mimic the Host Defense System.

**Supplementary Figures**

**Figure S1.** The Top 25 Annotation Results of Species-Specific Ortholog Families in the Plant-Parasitic Nematodes.
